# A Green Bridge: Enhancing a Multi-Pesticide Test for Food by Phase-Transfer Sample Treatment Coupled with LC/MS

**DOI:** 10.3390/molecules28196756

**Published:** 2023-09-22

**Authors:** Shaoming Jin, Yi Shen, Tongtong Liu, Ruiqiang Liang, Xiao Ning, Jin Cao

**Affiliations:** 1 Key Laboratory of Food Quality and Safety for State Market Regulation, National Institute of Food and Drug Control, Beijing 100050, China; myjackyming@126.com (S.J.);; 2 Center for Food Evaluation, State Administration for Market Regulation, Beijing 100070, China

**Keywords:** phase-transfer purification, QuEChERS, carrageenan, carbamate pesticides

## Abstract

The preparation and treatment of the sample has become an important part of the determination process, which directly affects the accuracy of detection. The preparation of the sample for final detection is actually a process of separation and transfer of the target to be tested from the sample matrix. The phase-transfer process of analysis and detection is the process of transferring the target substance to be measured from a complex multiphase system to a simple homogeneous system. This study shows a new phase-transfer process for food sample pretreatment in the determination of carbamate pesticides. Edible gum, xanthan gum, carrageenan, and gelatin were selected for purification testing from the perspective of eco-friendliness and safety. Phase-transfer purification process research was carried out on spinach and other foods. Compared with the commonly used QuEChERS method, the LC/MS results indicate that the straightforward carrageenan treatment process can significantly diminish the detection matrix effect and yield similarly superior detection parameters. The phase-transfer purification method with carrageenan has similar sensitivity and systematic error. The limits of detection and limits of quantitation of each pesticide compound in six plant sample substrates were 0.02–0.36 μg/kg and 0.06–1.9 μg/kg, respectively, which were lower than the residue limits here and abroad. Supplemental recoveries in six blank samples at 5, 20, and 100 μg/kg with the phase-transfer process method were better than those for the QuEChERS method. Positive determination results of actual samples using carrageenan phase-transfer purification proved that this method can be used for related detection from a practical point of view.

## 1. Introduction

Samples for analysis and detection often have a complex matrix, which coexists with the target substance to be measured, and the co-existing matrix substance will interfere with the detection of the target substance during the determination process, resulting in deviation of the result. Therefore, the preparation and treatment of the sample has become an important part of the detection, and it directly affects the accuracy of the determination. This effect is particularly serious when there are multiple detection targets in the sample and there are certain concentration differences among the multiple detection targets. From the point of view of accurate measurement, the matrix interference co-existing with the target to be detected in the sample is an important aspect of the late detection deviation, that is, the deviation of the detection signal [1,2,3]. The purpose of sample pretreatment is also to remove the relevant coexisting matrix in the fully extracted sample extract so that the detection environment of the final target matches the uncomplicated environment of the corresponding reference substance and the traceable value of the target substance in the sample can be obtained by comparison [4,5]. In recent years, many QuEChERS methods have been used for the detection of multi-pesticide residues, solvent extraction, dehydration, desorption with matrix, salting out, and removal of matrix by adsorption. With the increase in applications and reference standards, a variety of related formulations for adjusting the extraction and purification ratio have been widely used [6,7,8,9,10]. However, at present, this simple and fast method still has some problems. First, it is not a green treatment method, and there is a large amount of wasted reagents and consumables in the treatment. Second, for the sample matrix, not all sample formulations have the corresponding universality, and there is selectivity for relevant substrates, such as in food samples. Even if it is only for plant foods and the same target substance to be detected, for the proportion of chlorophyll, oil, protein, and water, it is necessary to adjust the extraction or purification formula.

How do we effectively extract, separate, and transfer the substance to be tested into a simple substrate environment through the coexistence of the matrix and the substance to be tested, and in the determination process, the standard substance with which it is compared in the same or similar detection environment? The approximate response results can be obtained so that the accuracy of the test results can be guaranteed and the reference with the standard material can be realized. The so-called phase-transfer process of analysis and detection is the process of transferring the target substance to be measured from a complex multiphase system to a simple homogeneous system. This is done to realize the premise assumptions of various detection methods in the process of establishing the detection principle in the detection. In order to achieve the removal of ineffective macro substances, the food industry should concentrate on obtaining nutrients as much as possible and then adopt corresponding homogenization or homogenization means to obtain relatively uniform food products [11,12]. For example, the use of flocculants [13], coagulants [14], edible glue [15], etc., in the process suggests that in the detection of food products, the corresponding green flocculation, edible glue enrichment, and other means can also be used for the phase transfer or purification of substances in the sample. For example, polyacrylamide is a commonly used synthetic polymer, which is often used in the sugar industry for flocculation to remove impurities in the virgin liquid and purify sugar [16]. Microbial flocculant common glycoproteins, mucopolysaccharides, proteins, cellulose, and other substances are used for the purification of drinking water and the removal of colored substances [17]. Edible gum is a kind of food additive. In meat processing, polysaccharide gum is commonly used to fix fat and starch to form homogeneous products. The use of xanthan gum jelly or candy, pigment, and polar substances, which can be evenly dispersed in the product, shows no phase separation [18]. Gellan gum, agar, carrageenan, and so forth in preserves, icing, jelly gel, yogurt, and other products play the role of stabilizer, dispersant, and thickening agent [19,20,21,22].

As organic chlorine pesticides are banned and the number of insect varieties resistant to organophosphorus insecticides is increasing, the amount of carbamate pesticides is increasing year by year, resulting in the residues of such pesticides in the environment and crops. Countries have formulated maximum residue limit (MRL) standards for carbamate pesticides [23]. Because of the structural characteristics of carbamate pesticides, the amino group is directly connected with the carbonyl group of carbamate. Hence, the polarity is strong and the thermal stability is poor, and it needs to be derived before it can be determined by gas chromatography (GC) [24]. Liquid chromatography–triple quadrupole tandem mass spectrometry is the preferred method for trace analysis because of its high sensitivity and good anti-interference ability [25,26]. In this study, on the basis of screening, common inorganic and organic flocculants, as well as edible gum, xanthan gum, carrageenan, and gelatin, were selected for purification testing from the perspectives of greenness and safety, as well as their physical and chemical properties. The test optimization was carried out on spinach and other foods. It was determined that in the detection of carbamate pesticide residues, carrageenan can be simply used to purify the sample extract solution. Compared with the commonly used QuEChERS method, the results show that the simple process of carrageenan treatment can effectively reduce the matrix effect of detection and obtain better detection parameters. It can be used as a method for the detection of carbamate pesticide residues in plant foods.

## 2. Results and Discussion

### 2.1. Selection of Food Substrates and Target Substances

The food matrix is complex, the pesticide residue is low, and the interference factors are many. The main reason is that the residual components are not easy to separate, enrich, and purify, and so the detection of relevant pesticides is not accurate. The extraction of target substances and the selection and optimization of substrate purification methods in sample pretreatment have become the key points of pesticide multi-residue detection. In 2003, Anastassiades and Lehotay proposed the original QuEChERS method [27]. Current QuEChERS methods include the original method, the American Society of Analytical Chemists standard method (AOAC 2007.01) [28], and the European Committee for Standardization standard method (EN 15662). According to the nature of the EN 15662 method, plant foods are divided into acid samples, high-water-content samples (water content ≥ 80%), low-water-content samples (water content < 80%), dry samples (grains), pigmentary samples, etc. According to the classification system, two pretreatment methods were used to treat the substrates of different plant foods. Specifically, there are six representative substrates, including acid samples (lemon, pH 3), high-water-content samples (apple and cabbage, 85–90% water content), low-water-content samples (banana, 70% water content), grain (rice, ≤10% water content), and pigment samples (spinach and chlorophyll). At the same time, according to the Maximum Residue Limits of Pesticides in Food (GB 2763-2021) [29], a total of 30 kinds of common carbamate pesticides and their active metabolites were selected with MRL standards.

### 2.2. Optimization of LC–MS/MS Conditions

In the positive ion mode of the electrospray ion source, a single standard solution of 30 kinds of carbamate pesticides was scanned to obtain stable parent ions, and the breakage voltage was optimized by SIM scanning mode. Two characteristic ion pairs with high response value were then selected for each compound as quantitative and qualitative ion pairs to further optimize the collision energy. The optimization results are shown in Table 1.

**Figure 1:**
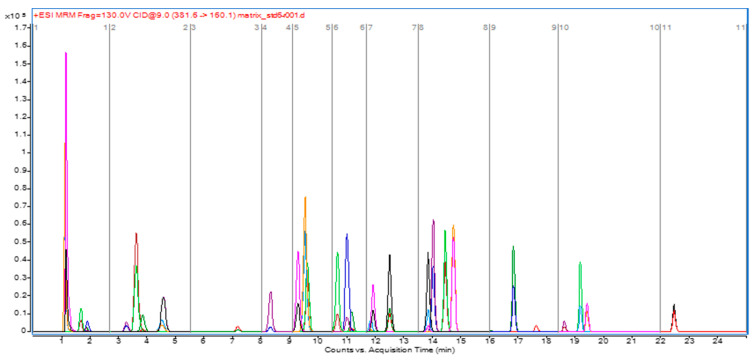
Extracted ion chromatogram of 30 carbamate pesticides.

At the same time, an Agilent Zorbax Eclipse XDB C18 column was compared with the Japanese Shiseido Type MG III column. They were subjected to testing using identical liquid phases, 0.1% (*v*/*v*) formic acid aqueous solution, and 0.1% (*v*/*v*) formic acid methanol solution. It was found that the separation degree and peak shape of aminocarb and propamocarb, which peaked first, were better when the latter was used, and the baseline drift was smaller. Therefore, a Shiseido Type MG III column was selected. In addition, the mobile phase systems with different compositions, NH_4_Ac (5 mmol/L)–acetonitrile, NH_4_Ac (5 mmol/L)–methanol, 0.1% (*v*/*v*) formic acid aqueous solution–0.1% (*v*/*v*) formic acid acetonitrile solution, and 0.1% (*v*/*v*) formic acid aqueous solution–0.1% (*v*/*v*) formic acid methanol solution, were compared. The results showed that when using acetonitrile, the peak shape of methiocarb was very poor, and the separation effect of 30 pesticides was not as good as that when the organic phase was methanol. The mobile-phase system of 0.1% (*v*/*v*) formic acid aqueous solution and 0.1% (*v*/*v*) formic acid methanol solution was more convenient to prepare and had good separation effect, and so we selected it as the mobile phase. Figure 1 shows the ion chromatogram for the extraction of 30 carbamate pesticide compounds in 100 μg/L mixed standard solution.

### 2.3. Optimization of Pre-Treatment Methods

#### 2.3.1. Optimization of Pre-Treatment Method 1

##### Selection of Purification Materials

Common flocculants include inorganic and organic chemicals, such as aluminum sulfate, magnesium sulfate, polyphosphate, and polyacrylamide. Because of certain safety problems or non-green factors, polysaccharide edible gum is mainly considered. In this study, xanthan gum, carrageenan, and gelatin were tested and selected. Xanthan gum is a water-soluble gum, which is the most characteristic of several microbial polysaccharides. It is also the largest and most widely used microbial polysaccharide in the world. Xanthan gum has good thickening, pseudoplastic rheology, water solubility, suspension, emulsion stability, acid and alkali resistance, salt resistance, temperature resistance, excellent compatibility, and other properties. It is widely used in the food industry [30]. Secondly, carrageenan also has good solubility. There are generally seven types, and the commonly used is κ-carrageenan. It can be dissolved in hot water and in cold water. Carrageenan and carrageenan sodium salt can also be dissolved, but the potassium salt and calcium salt of carrageenan can only absorb water and expand and cannot be dissolved. The gel formed by carrageenan is thermally reversible; that is, when heated, it condenses and melts into a solution, and when the solution is cooled, it forms a gel [31]. Edible gelatin consists of white or light-yellow transparent to translucent brittle flakes, particles, or powder that are lustrous, odorless, tasteless, and insoluble in cold water, ethyl ether, ethanol, chloroform, soluble in hot water, glycerin, acetic acid, salicylic acid, phthalic acid, urea, thiourea, thiocyanate, potassium bromide, and other solutions [32,33]. The relative density is l.3–1.4, which can slowly absorb 5–l0 times of cold water and expand and soften. When it absorbs more than twice the water, it is heated to 40 °C and melted into sol, and it forms a soft and elastic gel after cooling.

In the process of the experiment, the shrinking state of the gel after the addition of different acid and base was compared, and it was found that after the addition of NaOH, the water solution into the gel would leak. That is, the distribution of the target to be tested during the extraction process in the gel and the water solution is uneven, while the gelatin naturally formed in the gelatin process. Aqueous solutions also produce an unstable distribution of the situation. The state of xanthan gum strongly depends on the concentration, and the effective working concentration range used for phase transfer is too narrow, so carrageenan was finally used for treatment.

##### Choice of Carrageenan Quantity

After the aqueous extraction reagent is added to the sample homogenate, the amount of glue added will directly affect the co-existing matrix (mainly protein, carbohydrate, and other macro substances in the sample) and the distribution of pesticide to be detected in the solution in the later stage. According to the characteristics and solubility of carbamate components, with 20% acetonitrile water as the extraction solvent, 0.1, 0.3, 0.5, 0.7, 0.9, 1.1, 1.3, and 1.5 g of carrageenan were added separately in a water bath at 50 °C in a volume of 20 mL. After cooling to room temperature, it can be seen that under the above added amount, a gel can eventually form, and it can still become a solution by reheating. But under 0.3 g, good phase separation cannot be obtained by centrifugation. Above 1.1 g, there is expansion, indicating that it may cause uneven distribution of solute of the gel phase and water phase. Then, in the range of 0.3–1.1 g, the purification test was carried out with spinach homogenate and apple homogenate. It can be seen that in the process of gel formation, pigments and some fine fiber substances gathered in the gel, and a light-colored and transparent solution could be obtained after centrifugation. We added oxamyl and ethiofencarb to form an overall concentration of 20 μg/L in spinach and apple substrates with a corresponding volume of 20 mL. After optimization, carrageenan was added to 1.0 g and 0.5 g, and the concentrations of the two substances in spinach purification solution were 19.2 and 19.4 μg/L, respectively, with decreasing rates of 4% and 3%, and 19.5 and 19.6 μg/L in the apple purification solution, respectively, indicating that the gel phase mainly removed the matrix components. The substance to be detected is always a relatively homogeneous state in the whole system. Similarly, similar measurements were made for other substrates, and the overall concentration decreased to less than 10% in the above range, as shown in Table 2. It indicates that the overall recovery rate loss is within the allowable range. The amount of optimization for final processing is described in Section 3.2.

#### 2.3.2. Optimization of Pre-Treatment Method 2

In the experiment, the purification agents PSA and C18 and their ratio were optimized in the QuEChERS step, and the adsorption effect of purification reagent on 30 kinds of carbamate pesticides was investigated. PSA, PSA + C18, and C18 adsorbents were added to 30 kinds of pesticide mixed standard solutions with 10 μg/L concentration, and their concentrations were determined. The results show that C18 adsorbents had strong adsorption effects on carboxypropyl sulfide and carboxybutylsulfide, and their concentrations decreased by 90% and 85%, respectively. Therefore, the use of PSA as an adsorbent was eventually determined for plant foods. For samples containing part of the fat, such as rice, the fat was removed by freezing. At the same time, the influence of the dosage of PSA adsorbent on the purification effect of the blank sample matrix extract was investigated. Finally, it was determined that when the dosage of PSA reached 25 g/L, the influence of the interference peak could be significantly reduced. The purification effect was not significantly improved when the dosage continued to increase. For example, the recovery rate of six kinds of pesticides, such as methanocarb, antiaphid, dioxocarb, indocarb, prothiocarb, and furameocarb, decreased (more than 10%). For dark vegetables, the amount of GCB was investigated. It is clear that at the amount of 40.5 g/L, the influence of pigment was eliminated, and the recovery rate was not greatly affected. For acidic foods such as lemons, the amount of alkali added is optimized to reduce the influence of some carbamate substances, such as dioxocarb and ethylthiobenzocarb, by adding 400 μL of 5 mol/L NaOH solution to stabilize the relevant pesticide substances. At the same time, the alkaline solution is also used in the gumming treatment. See Section 3.2 of Materials and Methods for details.

### 2.4. Matrix Effect

The matrix effect will affect the repeatability, sensitivity, and accuracy of the analysis method. It is more prominent when the electrospray source is used, which mainly shows the ion inhibition effect on the target compound [34,35]. The matrix effect is the ratio of the slope of the matrix matching calibration curve to slope of the solvent standard calibration curve. The closer the ratio is to 1, the smaller is the matrix effect, and vice versa. The matrix effects of the six samples are shown in Table 3. The results showed that spinach had the largest matrix effect, followed by lemon. The basic effect of methanocarb and carbosultiocarb was the most obvious. The influence of matrix effect is often reduced by preparing a matrix matching standard curve, adding analytical protective agent and salting out. The method of matrix matching standard curve is used in this study.

### 2.5. Methodological Evaluation

#### 2.5.1. Standard Curve and Detection Limit

According to the MRLs of Pesticides in Food (GB 2763-2021), the maximum residue range of 30 target carbamate pesticides (50–5000 μg/kg) is limited. Three concentration levels, one order of magnitude higher, one order of magnitude lower, and the same order of magnitude lower than the MRLs, were selected for investigation. Combined with the response values of each pesticide and the saturation effect of the instrument, the matrix matching mixed standard solutions of 1, 2, 5, 10, 20, 50, 100, and 200 μg/L were prepared. The linear relationship of the pesticide compounds was good, in the range of 1–100 μg/L (equivalent to 1–100 μg/kg). In the range of 1–200 μg/L, the linearity of 17 pesticide compounds, such as aminocarb and propamocarb, was poor, and better correlation coefficients could be obtained by fitting the quadratic equation, which may be due to the saturation effect of the detector or ion source [13]. Considering that the quantitative accuracy of the quadratic curve equation is not high and that the chromatographic peak shape will deteriorate when the sample concentration is too high, the maximum mass concentration of the linear curve is set at 100 μg/L. The samples with higher pesticide residues need to be diluted for detection. The linear correlation coefficients of the matrix matching curves obtained by the two treatment methods for six plant samples are all greater than 0.998. The limit of detection (LOD) and limit of quantization (LOQ) of each pesticide compound in six plant sample substrates were determined by 3 times signal-to-noise ratio and 10 times signal-to-noise ratio, which were 0.02–0.36 μg/kg and 0.06–1.9 μg/kg, respectively, which were lower than the requirements of residue limits here and abroad. The slope and intercept of the linear curve and the recovery rate at three concentration levels were compared by using the two treatment methods. The comparison values are shown in Table 4.

The results show that phase-transfer purification method 1 with carrageenan has better sensitivity and smaller systematic error.

#### 2.5.2. Precision and Recovery Rate

For the six blank samples of banana, lemon, apple, Chinese cabbage, spinach, and rice, three levels of supplemental recovery tests were carried out. The spiking standard solutions were added into the homogenized sample solution before the purification process. The supplemental levels were 5, 20, and 100 μg/kg. Six parallel experiments were conducted for each level, and the matrix matching standard curve was quantitative. The results are shown in Table 5. 

The corresponding ratio of carrageenan purification is shown in Table 4. It shows that there is a better recovery rate at the three levels.

### 2.6. Determination of Actual Samples

After the method was established, seven batches of spinach, eight batches of apples, five batches of cabbage, five batches of bananas, three batches of rice, and three batches of lemons were determined by treatment methods 1 and 2. It was found that there were residues in the samples of spinach, apple, and Chinese cabbage, but none of them exceeded the national MRLs.

## 3. Materials and Methods

### 3.1. Instruments and Reagents

We used a 1290 Infinity liquid chromatograph with a 6460 Triple quadrupole Series Mass Spectrometer (Agilent, Santa Clara, CA, USA), a Milli-Q Pure water meter (Millipore Company, Burlington, MA, USA), a CF 16RX II centrifuge (Hitachi Corporation, Tokyo, Japan), an XHF-D high speed disperser (Ningbo Xinzhi Biotechnology Co., Ltd., Ningbo, China), an XP205 analytical balance (1 in 100,000), and an AL204 analytical balance (1 in 10,000) (Mettler, Greifensee, Switzerland).

Ethylenediamine-*n*-propyl silane (PSA) adsorbent: 40–60 μm particle size (Tianjin Bonaigel Technology Co., Ltd., Tianjin, China). Graphitized carbon black (GCB) and C18 adsorbent: 40 μm particle size (Agilent Corporation). Sodium citrate and NaCl were highly pure, and anhydrous MgSO_4_ was analytically pure (Sinophosphoric Chemical Reagents Co., Ltd., Ningbo, China). Disodium hydrogen citrate was analytically pure (Tokyo Chemical Industry Co., Ltd., Tokyo, Japan). Xanthan gum, carrageenan, and gelatin were purchased from Sinopharm Group Chemical Reagent Co., Ltd., Shanghai, China. Methanol and acetonitrile were chromatographically pure (Thermo Fisher Company, Waltham, MA, USA). Formic acid was analytically pure (Fluka Corporation, Everett, WA, USA). High-purity water was used in the experiment. Metolcarb, propoxur, carbofuran, bendiocarb, carbaryl, ethiofencarb, thiofanox, thiocarb, isoproarb, 2,3,5-trimethacarb, fenobucarb, diethofencarb, methiocarb, promecarb, fenoxycarb, indoxacarb, benfuracarb, furathiocarb, and carbosuifan pesticide control products were purchased from Dr. Ehrenstorfer Company in Germany. Aminocarb, propamocarb, aldicarb sulfoxide, aldicarb sulfone, oxamyl, thiofanox sulfoxide, pirimicarb, thiofanox sulfone, 3-hydroxycarbofuran, dioxacarb, and aldicarb were purchased from Sigma-Aldrich Company in the United States. Detailed information on these 30 pesticides are shown in Table 6.

The standard reserve solution of each pesticide with a mass concentration of 1 g/L was prepared with acetonitrile, and the mixed standard solution of 30 kinds of aminomethylate pesticides with a mass concentration of 5 mg/L was prepared with acetonitrile and stored at −18 °C.

Matrix matching standard solution: 2 mL of 7 blank sample extracts were separately transferred into a 15 mL centrifuge tube and gently dried with nitrogen (N_2_), and 2 mL of mixed standard solutions with mass concentrations of 1, 2, 5, 10, 20, 50, and 100 μg/L were added to prepare a series of matrix matching control solutions (ready to use). The blank sample was obtained through filtration using a 0.22 μm microporous membrane for analysis. See pre-treatment methods 1 and 2 below.

### 3.2. Sample Pretreatment Method

Pre-treatment method 1: Different types of samples were homogenized or ground according to their characteristics during pre-treatment. Apple, lemon, and cabbage were cut into pieces and then packaged and stored at −18 °C, respectively. Spinach was chopped, subpackaged, and then stored at −18 °C. Banana was chopped, subpackaged, and stored at −18 °C. Prior to the determination, homogenization was carried out immediately after removal from the refrigerator. All samples were not peeled when cut or chopped. Rice samples were subpackaged after crushing and stored at −18 °C. About 10.00 g of homogenized sample or crushed rice was weighed into a 50 mL plugged centrifuge tube, 20 mL 20% acetonitrile aqueous solution was added, and carrageenan of different weights was added. The weight of carrageenan added was related to the characteristics of the samples. For apple, cabbage, lemon, and rice, the additive amount was 0.3 g; for spinach, the additive amount was 1.0 g; and for banana, the additive amount was 0.6 g. After ultrasonic treatment at 50 °C, it was cooled to room temperature for 5 min and centrifuged at 8000 rpm for 10 min, and the supernatant was taken. It was filtered for detection by using a 0.22 μm microporous filter membrane.

Pre-treatment method 2: The homogenized or ground sample procedure was the same as in Method 1. About 10.00 g of homogenized sample or crushed rice was weighed into a 50 mL plugged centrifuge tube, and 20 mL of different solvents were added according to their characteristics. The mixture was shaken vigorously for 1 min. For apple, cabbage, and spinach, the added solvent was acetonitrile. For banana, the added solvent was 10 mL of acetonitrile and 8 mL of ice water. For crushed rice, the added solvent was 10 mL of acetonitrile and 20 mL of ice water. After that, we added 4 g of anhydrous MgSO_4_, 1 g of NaCl, 1 g of sodium citrate, and 0.5 g of disodium hydrogen citrate, shook it vigorously for 1 min, and centrifuged it at 8000 rpm for 5 min. It should be noted that lemon samples had special circumstances where an additional 0.4 mL of 5 mol/L NaOH solution was needed to be added before shaking. We took 6 mL of the upper solution and poured it into a 15 mL plugged centrifuge tube pre-filled with 150 mg PSA and 900 mg of anhydrous MgSO_4_. For spinach, 80 mg of GCB needed to be added to absorb the pigment. We swirled it for 30 s and centrifuged it at 5 000 rpm for 2 min. We took 4 mL of the supernatant and added 40 μL of 5% (*v*/*v*) formic acid acetonitrile solution. It was filtered for analysis by using a 0.22 μm microporous filter membrane.

### 3.3. LC–MS/MS Conditions

LC conditions: Shiseido Type MG III column (150 mm × 2.0 mm, 5 μm), column temperature, 35 °C; injection volume, 2 μL; flow rate, 0.3 mL/min. Mobile phase: phase A is 0.1% (*v*/*v*) formic acid aqueous solution, and phase B is 0.1% (*v*/*v*) formic acid methanol solution. Gradient elution procedure: 0–3 min, 35% B; 3–20 min, 35% B–90% B; 20–25 min, 90% B. The running time was 25 min.

MS conditions: Electrospray ion (ESI) source in positive ion mode. Drying temperature, 330 °C; flow rate, 8 L/min; atomizing gas pressure, 30 psi; sheath temperature, 250 °C, flow rate, 11 L/min; capillary voltage, 3500 V. Scanning mode: segmented multi-response monitoring mode. 

## 4. Conclusions

In this study, six representative plant sample substrates were selected to optimize a more green and environmentally friendly phase-transfer purification method, which was used for actual sample detection. Compared with the commonly used QuEChERS method, the results showed a more sensitive and accurate result, which also confirmed that materials closer to food can be used for relatively green inspection in food inspection. The limitations of this new purification process should also be taken into account. The target analyte of this study is carbamate pesticides, and the adsorption effect of carrageenan on them can be negligible. For other types of analytes, when using carrageenan for sample pretreatment, it is also necessary to consider the adsorption effect on the analyte, especially for polar compounds. However, this study does offer an avenue for the investigation of the corresponding low-carbonization approach.

## Figures and Tables

**Table 1:** LC–MS/MS parameters of the 30 carbamate pesticides.

Pesticides	Relative Retention Time **	Transitions (*m*/*z*)	Dewell Time (mS)	Fragmentor (V)	CE (eV)
aminocarb	0.06	209.3/152.1 *, 209.3/137.1	100	70	9, 25
propamocarb	0.06	189.2/102.1 *, 189.2/144.1	100	70	13, 9
aldicarb sulfoxide	0.11	229.1/166.1 *, 229.1/109.1	60	70	5, 13
aldicarb sulfone	0.13	245.1/166.1 *, 245.1/109.1	60	60	13, 17
oxamyl	0.13	242.1/72.1 *, 242.1/121.2	60	55	17, 9
thiofanox sulfoxide	0.26	257.1/200.0 *, 257.1/137.2	40	65	5, 13
pirimicarb	0.31	239.3/72.1 *, 239.3/182.1	40	65	20, 13
thiofanox sulfone	0.31	273.1/216.1 *, 273.1/137.1	40	65	9, 21
3-hydroxycarbofuran	0.38	238.4/163.1 *, 238.4/181.1	40	100	9, 5
dioxacarb	0.38	224.2/167.1 *, 224.2/123.1	40	40	5, 13
aldicarb	0.63	213.1/89.1 *, 213.1/116.1	200	75	13, 9
metolcarb	0.73	166.2/109.1 *, 166.2/94.1	200	30	9, 35
propoxur	0.82	210.2/111.0 *, 210.2/168.3	60	30	9, 4
carbofuran	0.85	222.3/165.1 *, 222.3/123.1	60	70	9, 21
bendiocarb	0.85	224.1/167.1 *, 224.1/109.1	60	70	5, 17
carbaryl	0.95	202.1/145.0 *, 202.1/127.3	60	40	5, 33
ethiofencarb	1.00	226.1/107.1 *, 226.1/164.1	60	50	5, 9
thiofanox	1.02	241.1/184.1 *, 241.1/57.2	60	60	5, 17
thiocarb	1.06	377.0/64.1 *, 377.0/113.0	60	120	13, 9
isoproarb	1.13	194.1/137.1 *, 194.1/95.1	60	60	5, 9
2,3,5-trimethacarb	1.13	137.1/122.0 *, 137.1/107.2	60	130	17, 25
fenobucarb	1.27	208.1/95.1 *, 208.1/152.1	40	60	9, 4
diethofencarb	1.29	268.2/226.1 *, 268.2/152.2	40	55	21, 5
methiocarb	1.31	226.1/169.1 *, 226.1/121.1	40	55	5, 17
promecarb	1.33	208.1/109.1 *, 208.1/105.1	40	50	5, 13
fenoxycarb	1.53	302.3/88.1 *, 302.3/116.1	100	100	17, 5
indoxacarb	1.70	528.1/150.2 *, 528.1/293.2	100	150	21, 9
benfuracarb	1.75	411.2/195.0 *, 411.2/252.1	100	90	21, 9
furathiocarb	1.80	383.2/252.1 *, 383.2/167.1	100	90	5, 25
carbosuifan	2.06	381.6/118.1 *, 381.6/160.1	200	130	13, 9

* Quantitative ion; ** relative retention time with reference to ethiofencarb.

**Table 2:** Recovery of oxamyl and ethiofencarb in six types of samples with different carrageenan purification, %.

Substrates	Pesticide	Carrageenan Addition/g in 20 mL				
		0.3	0.5	0.7	0.9	1.1
Banana	Oxamyl	95.2	97.6	97.1	96.3	95.9
	Ethiofencarb	94.2	96.1	96.3	95.4	95.4
Lemon	Oxamyl	95.3	97.4	97.1	97.1	96.5
	Ethiofencarb	96.4	98.2	97.7	97.5	96.4
Apple	Oxamyl	95.4	97.5	97.2	97.1	96.5
	Ethiofencarb	94.3	98	97.9	96.4	96.2
Spinach	Oxamyl	91.4	94	94.1	95.2	96.3
	Ethiofencarb	92	93.1	95.7	96.4	96.8
Cabbage	Oxamyl	96.7	98.2	98	97.4	97.1
	Ethiofencarb	95.7	97.8	97.1	96.4	96.4
Rice	Oxamyl	97.9	97.2	96.5	96.5	95.9
	Ethiofencarb	98.1	97.2	97.1	96.8	96.1

**Table 3:** Matrix effects of 30 pesticides in six types of samples compared by using two treatment methods.

Pesticide	Banana		Lemon		Apple		Spinach		Cabbage		Rice	
	M1 *	M2 **	M1 *	M2 **	M1 *	M2 **	M1 *	M2 **	M1 *	M2 **	M1 *	M2 **
aminocarb	0.8	0.6	0.7	0.3	0.8	0.7	0.7	0.5	0.8	0.7	0.9	0.9
propamocarb	1.1	0.9	0.8	0.7	0.9	0.9	0.8	0.7	0.9	0.9	0.8	0.8
aldicarb sulfoxide	1.1	1.1	0.9	0.7	0.9	1.1	0.7	0.7	0.8	0.7	0.9	0.9
aldicarb sulfone	1.1	1.1	0.9	0.8	0.9	0.9	0.7	0.6	0.9	0.9	0.9	0.9
oxamyl	1.1	1.1	0.9	0.9	0.9	0.9	0.7	0.7	0.9	0.9	0.9	0.9
thiofanox sulfoxide	1.1	1.1	0.9	0.8	0.9	0.9	0.7	0.8	0.9	0.9	0.9	0.9
pirimicarb	1.1	1.2	0.8	0.6	0.9	0.9	0.8	0.7	0.9	0.9	0.9	0.9
thiofanox sulfone	1.1	1.1	0.8	0.7	0.9	0.9	0.8	0.7	0.9	0.9	0.9	0.9
3-hydroxycarbofuran	1.1	1.1	0.9	0.8	0.9	0.9	0.8	0.7	0.7	0.6	0.9	0.9
dioxacarb	1.1	1.1	0.7	0.7	0.9	0.9	0.7	0.6	0.9	0.9	0.8	0.8
aldicarb	1.1	1.1	0.9	0.8	0.9	0.9	0.7	0.6	0.9	0.9	0.8	0.6
metolcarb	1.1	1.1	0.8	0.8	0.9	0.9	0.7	0.6	0.9	0.9	0.9	0.9
propoxur	1.1	1.1	0.8	0.7	0.9	0.9	0.8	0.5	0.9	0.9	0.9	0.9
carbofuran	1.1	1.2	0.8	0.7	0.9	0.9	0.7	0.5	0.9	0.9	0.7	0.7
bendiocarb	1.1	1.1	0.8	0.8	0.9	0.9	0.7	0.5	0.9	0.9	0.8	0.8
carbaryl	1.1	1.2	0.8	0.9	0.9	0.9	0.7	0.5	0.9	0.9	0.8	0.8
ethiofencarb	1.1	1.1	0.8	0.7	0.9	0.9	0.7	0.4	0.9	0.9	0.8	0.7
thiofanox	1.1	1.2	0.8	0.7	0.9	0.9	0.6	0.5	0.9	0.9	0.7	0.7
thiocarb	1.2	1.3	0.9	0.8	0.9	0.8	0.8	0.8	0.9	0.9	0.9	0.9
isoproarb	1.1	1.1	0.8	0.8	0.9	0.9	0.7	0.6	0.9	0.9	0.9	0.9
2,3,5-trimethacarb	0.9	1.1	0.8	0.6	0.9	0.8	0.7	0.6	0.9	0.9	0.7	0.7
fenobucarb	1.1	1.1	0.7	0.5	0.8	0.8	0.5	0.3	0.7	0.6	0.8	0.6
diethofencarb	1.1	1.1	0.9	0.7	0.9	0.9	0.9	0.9	0.8	0.6	0.8	0.8
methiocarb	1.1	1.1	0.9	0.7	0.9	0.9	0.5	0.3	0.7	0.7	0.8	0.8
promecarb	1.1	1.1	0.8	0.7	0.9	0.9	0.6	0.4	0.7	0.7	0.9	0.9
fenoxycarb	1.1	1.1	0.8	0.7	0.9	0.9	0.6	0.3	0.9	0.9	0.8	0.8
indoxacarb	1.1	0.9	1.1	1.2	0.9	0.9	0.6	0.5	0.9	0.9	0.7	0.6
benfuracarb	1.1	0.9	0.9	0.7	0.9	0.9	0.8	0.7	0.7	0.6	0.6	0.5
furathiocarb	0.9	0.9	0.8	0.8	0.9	0.9	0.7	0.6	0.9	0.9	0.8	0.8
carbosuifan	0.9	0.9	0.8	0.6	0.9	0.9	0.7	0.6	0.8	0.8	0.9	0.9

* Sample treatment with carrageenan purification; ** sample treatment with QuEChERS.

**Table 4:** Comparison of regression and recovery rates between treatment methods 1 and 2 *.

Pesticide	Rate of Linear Slope	Rate of Intercept	Comparison of Recovery		
			5 µg/kg	20 µg/kg	100 µg/kg
aminocarb	1.2	0.5	1.05	1.01	1.03
propamocarb	1.1	0.4	1.04	1.04	1.01
aldicarb sulfoxide	1.2	0.5	1.05	1.03	1.01
aldicarb sulfone	1.2	0.3	1.03	1.08	1.02
oxamyl	1.1	0.6	1.06	1.01	1.02
thiofanox sulfoxide	1.1	0.7	1.07	1.03	1.03
pirimicarb	1.3	0.5	1.05	1.05	1.04
thiofanox sulfone	1.2	0.4	1.04	1.02	1.01
3-hydroxycarbofuran	1.1	0.4	1.04	1.03	1.02
dioxacarb	1.2	0.3	1.03	1.04	1.01
aldicarb	1.1	0.4	1.04	1.01	1.01
metolcarb	1.1	0.3	1.03	1.02	1.07
propoxur	1.2	0.3	1.03	1.02	1.05
carbofuran	1.3	0.3	1.03	1.06	1.01
bendiocarb	1.2	0.4	1.02	1.04	1.01
carbaryl	1.2	0.5	1.04	1.05	1.04
ethiofencarb	1.2	0.3	1.03	1.02	1.01
thiofanox	1.2	0.3	1.03	1.01	1.06
thiocarb	1.1	0.4	1.02	1.04	1.01
isoproarb	1	0.2	1.02	1.02	1.04
2,3,5-trimethacarb	1	0.2	1.02	1.03	1.05
fenobucarb	1.1	0.4	1.04	1.01	1.02
diethofencarb	1.2	0.3	1.03	1.01	1.01
methiocarb	1.1	0.3	1.03	1.04	1.03
promecarb	1.2	0.3	1.03	1.02	1.04
fenoxycarb	1.1	0.3	1.03	1.01	1.01
indoxacarb	1.1	0.5	1.05	1.04	1.05
benfuracarb	1.1	0.5	1.05	1.03	1.02
furathiocarb	1.3	0.5	1.05	1.01	1.01
carbosuifan	1.2	0.5	1.05	1.03	1.03

* Treatment methods 1 and 2 refer to carrageenan purification and QuEChERS.

**Table 5:** The recoveries of low, medium, and high levels for six matrixes and RSD ranges.

Matrix	Supplemental Level			RSD Range
	5 μg/kg	20 μg/kg	100 μg/kg	
banana	59.84–127.06%	60.08–114.06%	56.13–110.05%	1.1–15%
lemon	62.05–91.61%	69.33–98.94%	71.83–107.02%	0.47–9.3%
apple	74.78–106.3%	60.51–125.2%	75.40–117.6%,	3.1–16%
cabbage	68.62–103.0%	69.81–99.31%	67.62–112.3%	1.2–13%
spinach	71.11–115.3%	77.45–125.3%	57.36–103.2%	3.2–14%
rice	85.70–106.0%	67.31–108.3%	70.70–97.61%	0.70–8.5%

**Table 6:** Detailed information on the 30 pesticides.

Pesticide	IUPAC Name	Formula
aminocarb	[4-(dimethylamino)-3-methylphenyl] N-methylcarbamate	C_11_H_16_N_2_O_2_
propamocarb	propyl N-[3-(dimethylamino)propyl]carbamate	C_9_H_20_N_2_O_2_
aldicarb sulfoxide	[(E)-(2-methyl-2-methylsulfinylpropylidene)amino] N-methylcarbamate	C_7_H_14_N_2_O_3_S
aldicarb sulfone	[(E)-(2-methyl-2-methylsulfonylpropylidene)amino] N-methylcarbamate	C_7_H_14_N_2_O_4_S
oxamyl	methyl (1Z)-2-(dimethylamino)-N-(methylcarbamoyloxy)-2-oxoethanimidothioate	C_7_H_13_N_3_O_3_S
thiofanox sulfoxide	[(Z)-(3,3-dimethyl-1-methylsulfinylbutan-2-ylidene)amino] N-methylcarbamate	C_9_H_18_N_2_O_3_S
pirimicarb	[2-(dimethylamino)-5,6-dimethylpyrimidin-4-yl] N,N-dimethylcarbamate	C_11_H_18_N_4_O_2_
thiofanox sulfone	[(3,3-dimethyl-1-methylsulfonylbutan-2-ylidene)amino] N-methylcarbamate	C_9_H_18_N_2_O_4_S
3-hydroxycarbofuran	(3-hydroxy-2,2-dimethyl-3H-1-benzofuran-7-yl) N-methylcarbamate	C_12_H_15_NO_4_
dioxacarb	[2-(1,3-dioxolan-2-yl)phenyl] N-methylcarbamate	C_11_H_13_NO_4_
aldicarb	[(E)-(2-methyl-2-methylsulfonylpropylidene)amino] N-methylcarbamate	C_7_H_14_N_2_O_4_S
metolcarb	(3-methylphenyl) N-methylcarbamate	C_9_H_11_NO_2_
propoxur	(2-propan-2-yloxyphenyl) N-methylcarbamate	C_11_H_15_NO_3_
carbofuran	(2,2-dimethyl-3H-1-benzofuran-7-yl) N-methylcarbamate	C_12_H_15_NO_3_
bendiocarb	(2,2-dimethyl-1,3-benzodioxol-4-yl) N-methylcarbamate	C_11_H_13_NO_4_
carbaryl	naphthalen-1-yl N-methylcarbamate	C_12_H_11_NO_2_
ethiofencarb	[2-(ethylsulfanylmethyl)phenyl] N-methylcarbamate	C_11_H_15_NO_2_S
thiofanox	[(3,3-dimethyl-1-methylsulfanylbutan-2-ylidene)amino] N-methylcarbamate	C_9_H_18_N_2_O_2_S
thiocarb	N,N-diethylcarbamodithioate	C_5_H_16_NNaO_3_S_2_
isoproarb	(2-propan-2-ylphenyl) N-methylcarbamate	C_11_H_15_NO_2_
2,3,5-trimethacarb	(2,3,5-trimethylphenyl) N-methylcarbamate	C_11_H_15_NO_2_
fenobucarb	(2-butan-2-ylphenyl) N-methylcarbamate	C_12_H_17_NO_2_
diethofencarb	propan-2-yl N-(3,4-diethoxyphenyl)carbamate	C_14_H_21_NO_4_
methiocarb	(3,5-dimethyl-4-methylsulfanylphenyl) N-methylcarbamate	C_11_H_15_NO_2_S
promecarb	(3-methyl-5-propan-2-ylphenyl) N-methylcarbamate	C_11_H_15_NO_2_S
fenoxycarb	ethyl N-[2-(4-phenoxyphenoxy)ethyl]carbamate	C_17_H_19_NO_4_
indoxacarb	methyl 7-chloro-2-[methoxycarbonyl-[4-(trifluoromethoxy)phenyl]carbamoyl]-3,5-dihydroindeno [1,2-e][1,3,4]oxadiazine-4a-carboxylate	C_22_H_17_ClF_3_N_3_O_7_
benfuracarb	ethyl 3-[[(2,2-dimethyl-3H-1-benzofuran-7-yl)oxycarbonyl-methylamino]sulfanyl-propan-2-ylamino]propanoate	C_20_H_30_N_2_O_5_S
furathiocarb	(2,2-dimethyl-3H-1-benzofuran-7-yl) N-[butoxycarbonyl(methyl)amino]sulfanyl-N-methylcarbamate	C_18_H_26_N_2_O_5_S
carbosuifan	(2,2-dimethyl-3H-1-benzofuran-7-yl) N-(dibutylamino)sulfanyl-N-methylcarbamate	C_20_H_32_N_2_O_3_S

## Data Availability

All data are included in this manuscript.

## Sample Availability

The plant food samples in the research were purchased from the supermarket and were detected as positive by testing. All processes are repeatable.
